# Influence of Intraocular Pressure on the Expression and Activity of Sodium–Potassium Pumps in the Corneal Endothelium

**DOI:** 10.3390/ijms251810227

**Published:** 2024-09-23

**Authors:** Princia Anney, Pascale Charpentier, Stéphanie Proulx

**Affiliations:** 1 Axe Médecine Régénératrice, Centre de Recherche du CHU de Québec-Université Laval, Québec City, QC G1S 4L8, Canada; princia-ahou-elsa.anney.1@ulaval.ca (P.A.); pascale.charpentier@crchudequebec.ulaval.ca (P.C.); 2 Centre de Recherche en Organogénèse Expérimentale de l’Université Laval/LOEX, Québec City, QC G1J 1Z4, Canada; 3 Département d’Ophtalmologie et ORL-Chirurgie Cervico-Faciale, Université Laval, Québec City, QC G1V 0A6, Canada

**Keywords:** corneal endothelium, Na+/K+-ATPase, tissue engineering, corneal bioreactor, intraocular pressure, flow

## Abstract

The corneal endothelium is responsible for pumping fluid out of the stroma in order to maintain corneal transparency, which depends in part on the expression and activity of sodium–potassium pumps. In this study, we evaluated how physiologic pressure and flow influence transcription, protein expression, and activity of Na+/K+-ATPase. Native and engineered corneal endothelia were cultured in a bioreactor in the presence of pressure and flow (hydrodynamic culture condition) or in a Petri dish (static culture condition). Transcription of *ATP1A1* was assessed using qPCR, the expression of the α1 subunit of Na+/K+-ATPase was measured using Western blots and ELISA assays, and Na+/K+-ATPase activity was evaluated using an ATPase assay in the presence of ouabain. Results show that physiologic pressure and flow increase the transcription and the protein expression of Na+/K+-ATPase α1 in engineered corneal endothelia, while they remain stable in native corneal endothelia. Interestingly, the activity of Na+/K+-ATPase was increased in the presence of physiologic pressure and flow in both native and engineered corneal endothelia. These findings highlight the role of the in vivo environment on the functionality of the corneal endothelium.

## 1. Introduction

Cellular monolayers that act as barriers between two spaces experience a wide range of external biomechanical forces, such as fluid shear stress and pressure. Mechanosensitive proteins participate in mechanotransduction cascades to translate these biomechanical signals into cellular responses, which then allow monolayers to maintain their structural integrity. Some of these mechanosensitive proteins are associated with intercellular junctions [1], ion channels [2,3], and cell–extracellular matrix receptors (integrins) [4].

In the eye, the corneal endothelium forms a monolayer of polarized cells, held together by adherens and tight junctions [5], restricting the entry of aqueous humor (from its apical side) into the corneal stroma (basal side). The apical and basolateral plasma membrane, of asymmetric composition, includes ion and fluid transporters, necessary to fulfill its function [6]. The main function of the corneal endothelium is to maintain stromal transparency. To do so, the corneal endothelium maintains the stroma in a partially dehydrated state (known as stromal deturgescence). Indeed, corneas in which the corneal endothelium has been removed become edematous and less transparent [7,8,9]. The corneal endothelium maintains stromal deturgescence thanks to the presence of numerous ionic pumps, such as Na+/K+-ATPases, while allowing entry of aqueous humor into the stroma to nourish the stromal keratocytes, which is possible by the presence of imperfect intercellular junctions. The functionality of the corneal endothelium therefore depends on a “pump–leak” balance, which is directly linked to the integrity of intercellular junctions and to the expression and activity of Na+/K+-ATPase pumps [10]. ATPases use the hydrolysis of ATP to drive the transport of cations against an electrochemical potential. Na+/K+-ATPase pumps establish and maintain high internal potassium and low internal sodium concentrations [11]. The resulting osmotic gradient then causes a passive pulling of water from the stroma into the anterior chamber. Na+/K+-ATPase pumps require a high amount of ATP to maintain stromal deturgescence, which is possible thanks to a high mitochondrial density in corneal endothelial cells (CECs). In fact, the corneal endothelium is one of the most metabolically active tissues in the body, second to photoreceptors [12]. Inhibition of Na+/K+-ATPase by ouabain creates stromal edema and clouding of the cornea [13].

The corneal endothelium is physiologically in constant contact with the biomechanical forces of intraocular pressure and liquid movement. The inflow of aqueous humor against its resistance to evacuation generates an intraocular pressure of 11 to 21 mmHg [14], and circulates at a speed of 2 to 5 µL/min [15]. We have previously demonstrated that these physiological hydrodynamic forces influence the transcription and cytolocalization of proteins associated with intercellular junctions, such as tight junctions, and that they had more influence on the endothelia whose junctions were forming (tissue-engineered corneal endothelia) than on endothelia with mature junctions (native corneal endothelia) [16,17]. Since there is a link between Na+/K+-ATPase and the formation of tight junctions [18], we postulated that mechanosensitive proteins that respond to pressure and flow will not only promote the expression and integrity of tight junctions [16,17], but also the expression and activity of Na+/K+-ATPase pumps.

## 2. Results

### 2.1. Gene Expression of ATP1A1 in Native and Tissue-Engineered Corneal Endothelium

**Figure 1:**
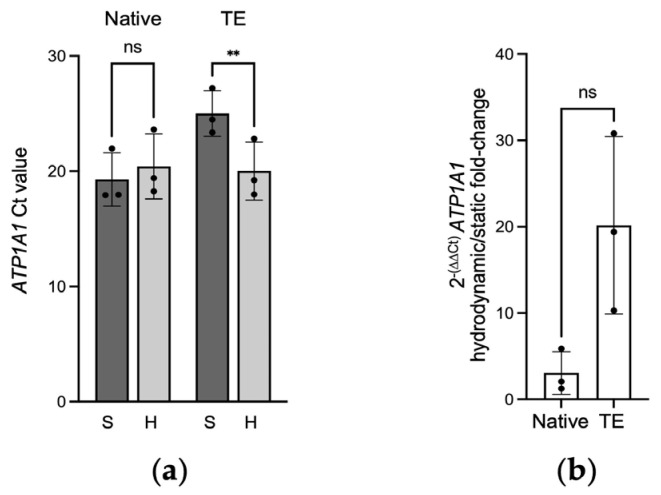
*ATP1A1* gene expression. (**a**) Threshold value (Ct) of *ATP1A1* mRNA of native and tissue-engineered (TE) endothelia cultured under static (S) or hydrodynamic (H) conditions. Results are normalized over B2M. Two-way ANOVA, ** *p* < 0.01, ns: not significant. (**b**) Hydrodynamic over static *ATP1A1* 2^−ΔΔCt^ fold change in native and tissue-engineered endothelia. Welch’s T test, ns: not significant. Results are presented as mean ± SD. *n* = 3, in triplicate.

Ct values are inversely proportional to gene expression. For native endothelia, the mean *ATP1A1* Ct values were similar for cells cultured under the static condition (19.3 ± 2.3) and those cultured under hydrodynamic conditions (20.4 ± 2.8) (*p* = 0.25) (Figure 1a), representing a gene transcription fold change of 3.1 ± 2.5 (Figure 1b). For the tissue-engineered endothelia, Ct values were 25.0 ± 2.0 and 20.0 ± 2.5 for the endothelia cultured under static and hydrodynamic conditions, respectively (Figure 1a). This increase in gene transcription was statistically significant (*p* = 0.0024). A 20.2 ± 10.3-fold increase in *ATP1A1* gene transcription was observed when the tissue-engineered endothelia were cultured under hydrodynamic conditions, compared to the static culture condition (Figure 1b).

### 2.2. Protein Expression of Na+/K+-ATPase α1 in Native and Tissue-Engineered Corneal Endothelium

**Figure 2:**
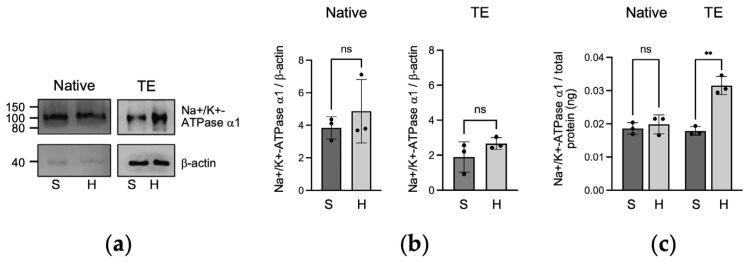
Na+/K+-ATPase α1 protein expression. (**a**) Representative Western blots of Na+/K+-ATPase α1 of native and tissue-engineered (TE) endothelia cultured under static (S) or hydrodynamic (H) conditions; (**b**) Western blots Na+/K+-ATPase α1 band intensity relative to β-actin, Welch’s T test, ns: not significant; (**c**) ELISA quantification of Na+/K+-ATPase α1 protein of native and tissue-engineered endothelia cultured under static or hydrodynamic conditions, normalized to total proteins. Welch’s T test ** *p* < 0.01, ns: not significant. Results are presented as mean ± SD. *n* = 3, in triplicate.

In the immunoblots analysis (Figure 2a,b), Na+/K+-ATPase α1 protein expression was similar between the static and the hydrodynamic culture conditions for both the native endothelia (*p* = 0.4638) and the tissue-engineered (*p* = 0.2611) corneal endothelium. Using a more sensitive ELISA assay (Figure 2c), Na+/K+-ATPase α1 protein expression was similar in the static and the hydrodynamic conditions for the native endothelia (*p* = 0.5644). However, a marked increase in protein expression was observed when the tissue-engineered corneal endothelia were cultured under hydrodynamic culture conditions (*p* = 0.0051).

### 2.3. Na+/K+-ATPase Activity in Native and Tissue-Engineered Corneal Endothelium

**Figure 3:**
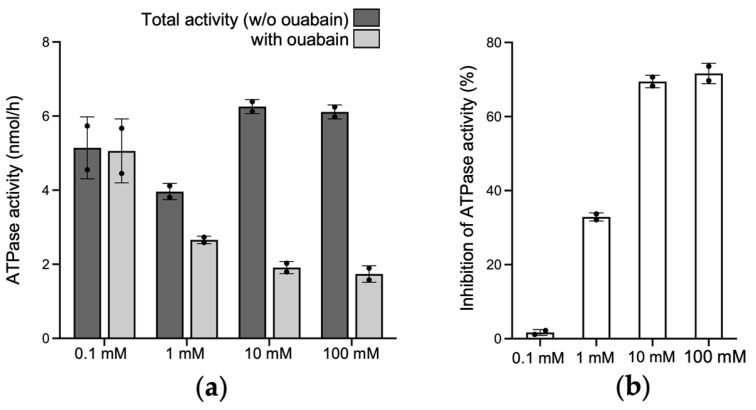
Ouabain dose–response assay. (**a**) ATPase activity in the absence and in the presence of different concentrations of ouabain; (**b**) percentage of inhibition of ATPase activity by different concentrations of ouabain. *n* = 1, in duplicate.

Ouabain is a specific Na+/K+-ATPase inhibitor. The ouabain dose–response assay revealed that the maximal Na+/K+-ATPase pump inhibition was achieved using 10 mM of ouabain (Figure 3). This concentration was thus used for the subsequent analysis.

**Figure 4:**
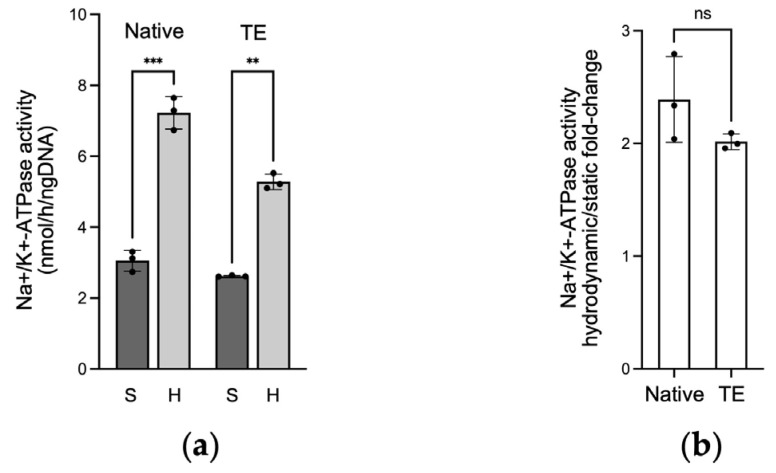
Na+/K+-ATPase activity (**a**) Na+/K+-ATPase activity of native and tissue-engineered (TE) endothelia cultured under static (S) or hydrodynamic (H) conditions. Results are standardized to total DNA. Two-way ANOVA, ** *p* < 0.01, *** *p* < 0.001; (**b**) hydrodynamic over static Na+/K+-ATPase activity fold change in native and tissue-engineered endothelia. Welch’s T test, ns: not significant. Results are presented as mean ± SD. *n* = 3, in triplicate.

Hydrodynamic culture conditions increased Na+/K+-ATPase activity for both the native and the tissue-engineered corneal endothelium, increasing from 3.1 ± 0.3 to 7.2 ± 0.5 nmol/h/ngDNA (fold change of 2.4 ± 0.4, *p* = 0.0004), and from 2.6 ± 0.02 to 5.3 ± 0.2 nmol/h/ngDNA (fold change of 2.0 ± 0.1, *p* = 0.0021), respectively (Figure 4).

## 3. Discussion

In this paper, we report that hydrodynamic culture conditions, namely physiological intraocular pressure and flow, generate distinct responses on sodium–potassium pump expression between native and tissue-engineered endothelia. In native tissues, the hydrodynamic condition did not influence *ATP1A1* transcription, nor did it influence the level of Na+/K+-ATPase α1 protein expression. However, it significantly increased its activity. In tissue-engineered corneal endothelium, culture under hydrodynamic conditions significantly increased *ATP1A1* transcription, protein expression, and Na+/K+-ATPase activity.

Our results confirm the well-documented presence of the α1 subunit of Na+/K+-ATPase in the corneal endothelium of native corneas [5,8,19,20,21,22]. In our experiments, pressure and flow did not influence Na+/K+-ATPase α1 protein expression in native tissues. This result is different from a previous paper where authors demonstrated a 4-fold increase in Na+/K+-ATPase α1 protein expression when native human corneas were stored in an active storage system that included intraocular pressure compared to those maintained in static organ culture [23]. The explanation for this difference with our results may reside in culture time, culture media, or temperature. Garcin and colleagues obtained their results following 28 days of culture in their bioreactor, corneas were cultured in CorneaMax, and the temperature was 31 °C [23], whereas in our experiments the corneas were maintained 3 days in the bioreactors, the culture media was Opti-MEM with supplements (see Section 4.2), and the temperature was 37 °C. Perhaps a longer culture would lead to a decreased Na+/K+-ATPase α1 expression in the static condition and maintain the expression in those cultured under hydrodynamic conditions, where we could then see an increase in expression between the two conditions. These could be interesting studies to perform in the future.

The expression of Na+/K+-ATPase α1 at both the transcriptomic and the proteomic levels in cultured CECs and in tissue-engineered corneal endothelium, under static culture conditions, has been previously shown [8,13,20,21,22,24,25]. Na+/K+-ATPase α1 expression following a short hydrodynamic culture is in accordance with a previous publication [26] where the expression of Na+/K+-ATPase α1 protein was observed in bovine CECs cultured in a perfusion system. However, they reported a similar level of expression between the perfused and the static cultures [26]. Since perfusion did not influence Na+/K+-ATPase expression [26], it could be postulated, in regard to our results that studied intraocular pressure and perfusion at the same time, that only the flow of culture media may not be sufficient to upregulate the expression of Na+/K+-ATPase α1 protein, and therefore that pressure could have more influence than flow.

The observation that native corneal endothelia were less influenced by hydrodynamic conditions compared to tissue-engineered endothelia may reside in the fact that native tissue possesses a mature corneal endothelial monolayer with well-formed intercellular junctions, which is a prerequisite for cell polarization and Na+/K+-ATPase expression [10], while tissue-engineered corneal endothelia are in the process of forming a functional monolayer with intercellular junctions that are still forming. We previously observed that hydrodynamic conditions increased the formation of tight junctions [16], as well as the transcription of *TJP1* [17], the gene coding for the tight junction protein ZO-1. Similar to what was observed at the intercellular junction level, the cues generated from pressure and flow may influence the maturation rate of the monolayer which in turn activates signaling pathways necessary for proper Na+/K+-ATPase α1 expression. In fact, we previously observed that the increase in *TJP1* transcription following hydrodynamic culture was more important in cells that had a less mature phenotype [17].

Stable physiologic intraocular pressure is important for proper ocular health as demonstrated by the fact that an elevated intraocular pressure in the anterior chamber is a significant risk factor for the development of glaucoma [27]. Chronic eye rubbing also increases intraocular pressure [28] and has been associated with the progression of keratoconus [29], while elevated intraocular pressure has been observed in Fuchs corneal endothelial dystrophy (FECD) [30]. Mechanosensitive ion channels, such as transient receptor potential cation channel subfamily V member 4 (TRPV4) [31] and Piezo1 [32], are postulated to be implicated in the development of glaucoma [33]. At the molecular level, activation of Piezo1 triggers Ca^2+^ influx, inducing Ca^2+^-dependent signaling cascades (reviewed in [33]), resulting in changes in gene expression [34], cytoskeletal structural alterations, and extracellular matrix remodeling [35]. For example, elevated Ca^2+^ has been shown to increase the transcription of *ATP1A1* in rat kidneys [36], and activation of Piezo1 has been reported to increase Na+/K+-ATPase activity in the mouse lens [37]. Given that Piezo1 is expressed in native human corneal endothelium [32], it could be expected that an increased hydrodynamic stimulation would affect CECs Na+/K+-ATPase activity. However, to the best of our knowledge, how an elevated intraocular pressure influences the expression or activity Na+/K+-ATPase in the corneal endothelium in health and disease has not been previously studied. Our corneal bioreactor could allow to address this question. Indeed, it would be possible to use tissue-engineered 3D models of eye diseases, for example FECD [22,38,39], place them in the corneal bioreactors under an elevated intraocular pressure condition, and study how this influences the expression and activity of Na+/K+-ATPase pumps.

Herein, corneas were placed in the corneal bioreactors for 3 days; however, it would be possible to place them for a longer period, thereby allowing for long-term effects of physiological or pathological intraocular pressure and/or flow. Apart from using a short-term culture condition, other limitations of this study include donor variability (age, post-mortem delays, method of conservation, …). Preliminary analysis on the influence of these variabilities did not show a correlation between them and the cell’s response to hydrodynamic culture; however, more corneas would be required in order to conclude on how age or delays affect *ATP1A1* transcription or protein expression following hydrodynamic culture. Compared to cells cultured on a rigid substrate, our study design has the advantage of studying CECs adhered on a substrate (native stroma) that can be stretched by pressure, allowing for a more physiologic cell response. This is particularly relevant considering that for cells cultured on plastic, the observed effects of hydrostatic pressure may be a consequence of changes in oxygen tension rather that pressure itself [40].

The knowledge from this study may be helpful for cornea preservation by eye banks. In America, eye banks store their corneas in a preservation media (Optisol), at 4 °C, in a static condition. In view of our results, adding a physiological pressure and flow on the endothelial side of the stored corneas may help to maintain a pool of functional Na+/K+-ATPase pumps in the corneas destined for grafting, which may improve the efficiency of stromal deturgescence once the tissue is grafted. Such a device has already been proposed in Europe [23]. Optimal preservation of corneas, especially regarding corneal endothelium functionality, is important in order to offer the patient the best corneas to treat their pathology.

## 4. Materials and Methods

### 4.1. Corneal Specimens

This study was conducted according to our institution’s guidelines (CHU de Québec-Université Laval, Québec, Canada) and the Declaration of Helsinki. The research protocol was approved by the ethics committee of the «Bureau de l’éthique de la recherche du CHU de Québec-Université Laval» (DR-002-1382). Twenty-seven pairs of healthy research-grade human corneas (see Table 1, Table 2 and Table 3 for tissue description), unsuitable for grafting, were obtained through the “Ocular tissues for vision research” infrastructure of the Vision Sciences Research Network, in collaboration with our local eye bank (Banque d’yeux du Centre universitaire d’ophtalmologie (CUO), CHU de Québec-Université Laval, Québec, Canada), with next-of-kin consent. 

### 4.2. Cell Culture and Tissue Engineering of the Corneal Endothelium

Isolation and culture of CECs were performed as previously described [41], using a peel and digest method. Cells were cultured in Opti-MEM I (Invitrogen, Burlington, ON, Canada), 0.2 g/L CaCl_2_ (Millipore-Sigma, Oakville, ON, Canada), 8% fetal bovine serum (HyClone, Logan, UT, USA), 5 ng/mL human epidermal growth factor (Austral Biologicals, San Ramon, CA, USA), 20 µg/mL ascorbic acid (Millipore-Sigma), 0.08% chondroitin sulfate (Millipopre Sigma) and 100 IU/mL penicillin/streptomycin G (Millipore-Sigma). Cells were routinely passaged at a cell density of 15,000–20,000 cells/cm^2^ and used between passages 1 and 9 (Table 1).

Tissue engineering of the corneal endothelium was achieved as previously described [20]. Native human corneas went through three freeze/thaw cycles and were stored at −20 °C until use (range 35 to 373 days, mean 116 ± 101; Table 1). On the day of the reconstruction, they were thawed, rinsed to remove the dead cells, and observed under a stereomicroscope (Nikon SMZ800, Mississauga, ON, Canada) to confirm the absence of cells. Confluent CECs were seeded on devitalized corneas at a density of 2500 cells/mm^2^ and cultured for 3 days before placing them under hydrodynamic or static culture conditions. The two corneas from the same donor were seeded with the same cell population.

### 4.3. Culture under Hydrodynamic and Static Conditions

One of the pair of either the tissue-engineered or the native cornea was placed in a corneal bioreactor (ECMTech Inc., Quebec City, Quebec, Canada) at a physiological pressure between 16 and 21 mmHg and a flow of 5 µL/min (hydrodynamic condition), for 3 days, while the other was cultured in a Petri dish (static condition), also for 3 days. Mounting and culturing of corneas in the bioreactor were performed as previously described [16,17].

### 4.4. Quantitative PCR (qPCR)

Total RNA was extracted from the corneal endothelium of native (*n* = 3) and tissue-engineered (*n* = 3) corneas cultured under hydrodynamic or static conditions, according to the manufacturer guidelines (Qiagen Biotechnology, Toronto, ON, Canada). RNA quantity was determined by an optical density measurement at 260 nm. cDNA was synthesized using oligo (dT) primers with reverse transcriptase (Brilliant III SYBR Master Mix; Agilent Technologies, Mississauga, ON, Canada). The cDNA samples were subjected to PCR with specific primers for *ATP1A1* (5′ CCAGAGGAGATCGCCGAAGC 3′ and 3′ GCGGTACGGTCTCGGAACT 5′). B2M gene served as an internal standard for sample normalization (5′ CCAGAGGAGATCGCCGAAGC 3′ and 3′ GCGGTACGGTCTCGGAACT 5′). Quantitative PCR was performed using a Rotor Gene Q real-time PCR Cycler (Qiagen), in triplicate. Synthesis of double-stranded DNA during PCR was monitored using Brilliant III UltraFast SYBR Green (Agilent Technologies).

### 4.5. Western Blotting Analysis

Proteins from the endothelium of native (*n* = 3) and tissue-engineered (*n* = 3) corneas, cultured under hydrodynamic or static conditions, were extracted using 100 µL of RIPA lysis buffer (Thermo Fisher Scientific, Mississauga, ON, Canada). The protein concentration was measured with a BCA assay kit (Fisher Scientific, Ottawa, ON, Canada). The samples were subjected to gel electrophoresis in 10% polyacrylamide gels and Western blotting was performed. Membranes were blocked with 5% milk in Tris-buffered saline. Primary antibodies (mouse anti-Na+/K+-ATPase α1, Millipore-Sigma; mouse anti-β-actin, Millipore-Sigma) were diluted 1:1000 and 1:2000, respectively, in 1% milk. The secondary horse anti-mouse antibody conjugated to peroxidase (New England Biolabs, Whitby, Ontario, Canada) was diluted 1:2000 in 1% milk. Bands were revealed using Western Sure Premium Chemiluminescent Substrate (LI-COR Biosciences, Lincoln, NE, USA) following a 4-min incubation in the dark, rinsed, then imaged using the C-Digit Chemiluminescence Western Blot Scanner (LI-COR Biosciences). β-actin was used as an internal loading control for protein normalization.

### 4.6. ELISA Analysis

The same proteins extracted for the Western blot analysis were used to measure the amount of Na+/K+-ATPase α1 by ELISA assay, following the manufacturer guidelines (ATP1A1 ELISA Kit, Aviva Systems Biology, San Diego, CA, USA). Fluorescence at 450 nm was measured using a microplate reader (SpectraMax ID3, Molecular Devices, San Jose, CA, USA). The results were normalized to the quantity of total proteins as assessed using BCA (see Section 4.5).

### 4.7. ATPase Assay

The ouabain dose–response assay was performed using native rabbit corneas (*n* = 4), obtained from a local slaughterhouse, within 24 h of death. ATPase activity was measured according to the manufacturer’s instructions (ATPase assay, Abcam, Cambridge, UK). A dose–response assay using 0.1, 1, 10, and 100 mM of ouabain octahydrate (Millipore Sigma) (N = 1 cornea per condition) was performed in order to determine the amount of ouabain for maximal ATPase inhibition. Assays were performed in triplicate.

ATPase activity of native (N = 3) and engineered (N = 3) corneal endothelium, in the presence and absence of 10 mM ouabain, was measured as described above, in triplicate. Na+/K+-ATPase activity was calculated as the difference in ATPase activity between cells exposed to ouabain and those that were not. The results were normalized with the total amount of DNA (New England Biolabs, Whitby, ON, Canada), as described by the manufacturer.

### 4.8. Statistical Analyses

Data are presented as mean ± standard deviation (SD). In all cases, “*n*” represents the number of populations. Statistical significance was analyzed using two-way ANOVA or Welch’s T test as specified in figure captions (Prism v. 9 software, GraphPad, San Diego, CA, USA) with a meaning threshold at *p* = 0.05.

## Figures and Tables

**Table 1:** Tissue information of corneas used for corneal endothelial cell isolation and culture.

No.	Age, Sex	Preservation	Delay between Death and Culture (days)	Passage	Figure
1	65, F	Optisol, 4 °C	30	4	Figure 1—PCR
2	58, F	Optisol, 4 °C	n/a	9	Figure 1—PCR
3	80, M	Fresh	2	6	Figure 1—PCR
4	64, M	Optisol, 4 °C	41	1	Figure 2—WB/ELISA
5	53, F	Optisol, 4 °C	41	1	Figure 2—WB/ELISA
6	85, F	Optisol, 4 °C	41	1	Figure 2—WB/ELISA
7	79, M	Optisol, 4 °C	26	3	Figure 4—ATPase activity
8	83, M	Fresh	7	1	Figure 4—ATPase activity
9	48, M	Optisol, 4 °C	5	2	Figure 4—ATPase activity

M, male; F, female.

**Table 2:** Tissue information of corneas that were devitalized for tissue engineering.

No.	Age, Sex	Preservation	Time Preserved at −20 °C (days)	Figure
10	72, F	Optisol, 4 °C	373	Figure 1—PCR
11	83, M	Optisol, 4 °C	113	Figure 1—PCR
12	77, F	Optisol, 4 °C	35	Figure 1—PCR
13	66, F	Optisol, 4 °C	106	Figure 2—WB/ELISA
14	65, M	Optisol, 4 °C	113	Figure 2—WB/ELISA
15	67, F	Optisol, 4 °C	91	Figure 2—WB/ELISA
16	76, M	Optisol, 4 °C	43	Figure 4—ATPase activity
17	74, F	Optisol, 4 °C	119	Figure 4—ATPase activity
18	70, M	Optisol, 4 °C	55	Figure 4—ATPase activity

M, male; F, female.

**Table 3:** Tissue information of native corneas used ex vivo.

No.	Age, Sex	Preservation	Delay between Death and Bioreactor (days)	Figure
19	70, M	Fresh	4	Figure 1—PCR
20	70, F	Fresh	5	Figure 1—PCR
21	56, F	Fresh	3	Figure 1—PCR
22	78, F	Optisol, 4 °C	28	Figure 2—WB/ELISA
23	84, F	Optisol, 4 °C	20	Figure 2—WB/ELISA
24	76, F	Optisol, 4 °C	9	Figure 2—WB/ELISA
25	79, M	Fresh	12	Figure 4—ATPase activity
26	64, F	Fresh	10	Figure 4—ATPase activity
27	80, M	Fresh	9	Figure 4—ATPase activity

M, male; F, female.

## Data Availability

The raw data supporting the conclusions of this article will be made available by the authors on request.
